# Double Anonymity and the Peer Review Process

**DOI:** 10.1100/tsw.2006.228

**Published:** 2006-10-09

**Authors:** Richard J.C. Brown

**Affiliations:** Analytical Science Group, National Physical Laboratory, Teddington, Middlesex, TW11 0LW, UK

**Keywords:** peer review, double anonymity, scientific journals

## Abstract

The process of peer review for submissions to scientific journals is a well-established and widely used procedure. Review by one's peers is a well-recognised and long-standing method of appraisal. Throughout all branches of science, medicine, humanities, art, literature, politics, sport, and in fact almost all areas of human endeavour, the judgement of work by an individual or group of experts in similar fields of study is the most rigorous and valuable form of recognition. “Peer review”, as this process is commonly known, is an important method of assuring quality, relevance and novelty of work. However, is there still room for improvement in the procedural aspects of peer review?

## INTRODUCTION

Review by one's peers is a well-recognised and long-standing method of appraisal. Throughout all branches of science, medicine, humanities, art, literature, politics, sport, and in fact almost all areas of human endeavour, the judgement of work by a individual or group of experts in similar fields of study is the most rigorous and valuable form of recognition[1]. "Peer review", as this process is commonly known, is an important method of assuring quality, relevance, and novelty of work. Not only is review by one's peers, the people whose work one is most likely to know and respect, the most valuable form of appraisal, but in most specialist subjects, it is only those who share the authors' specialty whom are able to robustly critique the work. Additionally, it is rare for authors to produce a perfect piece of work and there is often opportunity for improvement that may stand out only to someone with special expertise or experience, who is examining the work from a different standpoint. Therefore, showing work to others increases the probability that weaknesses will be identified and corrected. The process also forces authors to meet the standards of their discipline. Publications that have not undergone peer review are likely to be regarded with suspicion by academics and professionals in most fields.

* This paper expresses the personal opinions of the author and does not necessarily represent the views of the National Physical Laboratory or NPL Management Limited.

Peer review has only been a universal requirement for publication in scientific journals since the middle of the 20$^{th}$ century[2]. Famously, the publication of Albert Einstein's *Annus Mirabilis* papers in *Annalen der Physik* in 1905, and Watson and Crick's 1951 paper on the structure of DNA in *Nature*, were not peer reviewed. However, as the number of submissions burgeoned and the complexity of work increased, peer review became a fundamental pillar for journals in order to establish credibility and maintain standards of academic rigour. It is nowadays common practice for the peer reviewers, or "referees", to remain anonymous to the author, whilst the referees know the authors' identity. This has been the accepted wisdom for many years, despite single anonymity peer review being less than transparent, but is there room for improvement in these processes?

## DISCUSSION

When I receive a request, almost always electronically, from a scientific journal to referee a manuscript, I always follow the same routine. On discovering the e-mail's purpose, I quickly click on the links that allow me to access and print the manuscript, but always omit to print the front page. Why this curious procedure? The reason is that I do not want to see who has written what I am about to read. I want to make entirely sure that knowledge of who the author, or authors, are does not affect how I read, interpret, or judge the manuscript, either consciously, or, I would like to think more likely, subconsciously. Am I alone in my fear of this potentially human frailty? Can any of us put our hand on our heart and truthfully say that on discovering the identity of the author(s) of a particular piece of work we are not provoked into having just the slightest opinion on its content? In everyday life, this is something that is not unusual; in fact, it may be very helpful. We often judge whether to spend our money, or invest our leisure time, based on how much we have liked or disliked the previous work of a particular novelist, actor, pop group, or crossword compiler! This type of judgement would obviously not be satisfactory in peer review. In extreme cases, lack of author anonymity has been known to result in discrimination on grounds of gender, race, or nationality[3], or even scientific fraud[4]. This is very rare and, of course, not a general criticism of the vast body of referees whose unpaid, unselfish devotion of their (spare) time to reviewing manuscripts, and proposals for funding, makes the academic world go round. Instead, it is a search to see if things can be made even better. Certainly, sociologists contend that reviewers may feel under pressure to more readily accept for publication the work of a well-known scientist[5]. Conversely, it is thought that reviewers may treat with more suspicion or scepticism the work of authors who are new to the field or have relatively few publications in the area. However, other scientific sociological studies have pointed out that there is a very large number of scientific journals in which work can be published, making any systematic abuse almost impossible. In addition, the decision-making process of peer review, in which each referee gives his opinions separately and without consultation with the other members, is intended to mitigate some of these problems, although it is not a solution.

There are arguments for[6] and against[7] anonymity on both sides of the peer review process and a balance clearly must be struck between ensuring the fairness of the process and upholding the robustness and quality of published literature. However these two requirements do not need to be mutually exclusive.

Four situations involving different anonymity states in peer review can be imagined. These are presented in Table 1. Analysis of Table 1 would suggest that a system of "no anonymity" would be an improvement to the current system. Both referees and authors would be directly associated with the comments they make and would have to be willing to justify them. Already, in some academic fields, journals offer the reviewer the option of waiving their anonymity. Moreover, published manuscripts sometimes contain acknowledgements to named referees who have assisted in improving the work, principally because they have made themselves known to the authors. However, this system may still be open to influence by some, and there is a strong argument that referees, who usually perform the task in their own time, for no payment, and are generally in short supply, should be protected. Some studies have shown that the introduction of open peer review does not improve the quality of the review, nor the time taken to review, but did have the effect of increasing the likelihood of reviewers declining to review. A

**TABLE 1**
**The Four "Anonymity States" in Peer Review**

| | | **Referee** | |
|---|---|---|---|
| | | **Anonymous** | **Known** |
| **Author** | **Anonymous** | **Double anonymity**. Work can only be judged on quality alone. The author is assured that his work has been fairly assessed. The referee is assured of protection. No possibility of referees imposing their own prejudices, and being influenced in either direction by the status of the author. Possibility of referee still hiding behind anonymity. The least open to abuse or influence. | **Reverse single anonymity**. The opposite to the current system. The referee is exposed to potential criticism from the author who is shielded by anonymity. Prejudices of the author for or against the referee may lead to a belief of unfair judgement. Arguably the lease satisfactory of the four scenarios. |
| | **Known** | **Single anonymity**. The current system. Possibilities of referees imposing their own prejudices, and being influenced in either direction by the status of the author, whilst still being shielded by anonymity. Author may believe they have been unfairly judged. Arguably the scenario most open to abuse. | **No anonymity**. Both parties are aware of each other's identity and there is no chance of hiding behind anonymity. However the referee is still exposed to criticism from the author, and may also be influenced in either direction by the status of the author. Prejudices of the author for or against the referee may lead to a belief of unfair judgement. Still an improvement on the current system. |

"no anonymity" approach may not be the best solution for peer review in the long term, although the voluntary waiving of anonymity by referees is to be encouraged, and this is currently being taken up by many journals.

It would seem that the time has possibly come to promote a "double anonymity" rationale for peer reviewing. This is the form of peer review least open to abuse, offering the greatest improvement to the current system. There should be no requirement for a referee to know the identity of the author as the work should be judged on content alone. There is still the possibility that referees may hide behind anonymity, but it is thought that the need to protect referees outweighs this concern. It seems that the strongest argument for double anonymity in peer review is that, for those in support of scientific rigour and fairness, there can be no credible reasons for arguing against its introduction! Implementing double anonymity means that a referee should not be unduly influenced by the status of the author or the author's previous publication record, and there should be no feelings of unfair judgements from the author.

## CONCLUSION

In my opinion, there is a way to improve, at least the perception of, fairness in peer review: to not know who the authors are — double anonymity[8]. The view was proposed that the author of a manuscript should remain anonymous to those doing the reviewing. There are other possibilities, of course, which differ from the status quo: no anonymity, where both the author and the referee are known to each other (this is already done by some journals on a voluntary basis), or the more unfeasible option where the referee is known, but the author remains anonymous, which seems to have few redeeming features! Of these, double anonymity seems the best option. It has the least possibility for bias or partiality, and it is difficult for anyone to argue in favour of needing to know the authorship of work that they are refereeing

— surely it couldn't allow one to do a *better* job? From the authors' point of view as well, this must be an improvement. Knowing that one is anonymous to those who are refereeing your work can only serve to increase the perception of fairness and impartiality.

Of course, for those scientific research areas that are very sparsely populated, for those referees who are extremely well connected and informed about current research in their area, or for authors whose style makes them instantly recognisable, despite double anonymity, it may still be possible to determine whose work they are refereeing. This problem is, of course, more difficult to overcome. It is up to editors in this instance to be more radical in their choice of referees to avoid these types of issues. Even if the large majority of current peer review is fair and free from bias, which is currently believed to be the case, double anonymity would still provide the additional benefit that it removes any possible perception of unfairness, even if unfairness does not exist. Is it time that scientific journals started examining this possibility seriously?
